# Clinical Behavior and Complications of Mandibular Full-Arch Fixed Dental Prostheses Supported by Three Dental Implants. A Systematic Review and Meta-Analysis

**DOI:** 10.3390/biology10040308

**Published:** 2021-04-08

**Authors:** Luis Sánchez-Labrador, Pedro Molinero-Mourelle, Jorge Cortés-Bretón Brinkmann, Juan Carlos Prados-Frutos, Miguel Gómez-Polo, José María Martínez-González

**Affiliations:** 1 Department of Dental Clinical Specialties, Faculty of Dentistry, Complutense University of Madrid, 28040 Madrid, Spain; luissanc@ucm.es (L.S.-L.); jmargo@odon.ucm.es (J.M.M.-G.); 2 Department of Conservative Dentistry and Orofacial Prosthodontics, Faculty of Dentistry, Complutense University of Madrid, 28040 Madrid, Spain; pedromol@ucm.es (P.M.-M.); mgomezpo@ucm.es (M.G.-P.); 3 Department of Reconstructive Dentistry and Gerodontology, School of Dental Medicine, University of Bern, 3010 Bern, Switzerland; 4 Department of Medical Specialties and Public Health, Rey Juan Carlos University, 28922 Madrid, Spain; prof.prados@gmail.com; 5 IDIBO GROUP (Group of High-Performance Research, Development and Innovation in Dental Biomaterials), Department of Medical Specialties and Public Health, Rey Juan Carlos University, 28922 Madrid, Spain

**Keywords:** dental implants, fixed prostheses, implant-supported prostheses, mandibular fixed prostheses

## Abstract

**Simple Summary:**

Traditionally, 4–6 implants have been regarded as the optimal number of implants for supporting mandibular implant-supported fixed complete dental prostheses (ISFCDP). However, several studies have evaluated the clinical behavior and complications of ISFCDPs on three implants considering this option as a potentially valid alternative treatment. Fewer implants will not only reduce the economic cost but can also shorten surgical treatment time and minimize trauma, which will encourage more patients to receive this type of treatment.

**Abstract:**

This systematic review and meta-analysis set out to assess the clinical behavior of mandibular implant-supported fixed complete dental prostheses (ISFCDP) on three dental implants by analyzing implant and prosthetic survival rates, marginal bone loss, biological/technical complications, and patient-reported outcomes. The review was conducted according to PRISMA guidelines. Electronic searches were conducted in the Medline (PubMed), Web of Science, and Cochrane databases, complimented by a manual search in specialist journals for relevant articles published up to February 2021. The Newcastle-Ottawa Quality Assessment Scale tool was used to assess the quality of evidence in the studies reviewed. The study included 13 articles with 728 patients treated with 2184 implants. A mean implant survival rate of 95.9% (95% CI: 94.6–97.3%) and a prosthetic survival rate of 97.0% (95% CI: 95.7–98.3%) were obtained over 1–6-year follow-up periods. Mandibular implant-supported fixed complete dental prostheses on three dental implants would appear to be a viable option for restoring the edentulous mandible in comparison with mandibular ISFCDP on more than three implants. Further comparative studies are needed, with adequate protocols, as well as sufficient sample sizes and follow-up periods to confirm these findings.

## 1. Introduction

The percentage of edentulous people increases with age [1], making edentulism a common disability among the aging population associated with anatomical, functional and psychological changes [2]. The most commonly used option for rehabilitation of edentulous patients has typically been the conventional mucosa-supported complete denture. Nevertheless, when placed in the mandible, this type of prosthesis can be unstable and uncomfortable [3], making it difficult to perform essential functions such as chewing and speaking [1]. For these patients, implant-supported fixed complete dental prostheses (ISFCDP) may offer improved chewing function and esthetics and contribute to a consistently improved quality of life [1]. At the same time, the ISFCDP will prevent posterior mandibular ridge resorption [2].

Traditionally, 4–6 implants have been regarded as the optimal number of implants for supporting an ISFCDP. However, in 1999 Brånemark et al. [4] published the first clinical study (treating 50 patients) of rehabilitations with ISFCDPs supported by only three implants. The authors achieved a mean implant and prosthetic survival rate of 98% over three years using the Novum protocol. Since then, several studies have evaluated the clinical behavior and complications of ISFCDPs on three implants [5,6,7,8,9,10,11,12].

The potential for reducing the number of implants placed depends on load distribution; anterior and posterior implants receive the greatest loads regardless of the implant number or position, so that there is no need for large numbers of supporting implants [1].

Moreover, a higher number of implants result in a more complicated and expensive rehabilitation phase in terms of prosthetic fabrication (i.e., implant-framework accuracy and passive fit) [13,14], and so is often refused by patients. In addition, fewer implants will not only reduce the economic cost but can also shorten surgical treatment time and minimize trauma, which will encourage more patients to receive this type of treatment [15,16].

Despite the advantages described in the literature [17,18], this treatment modality requires specific conditions and rigid clinical characteristics [19,20]. In this sense, anatomical conditions play an important role due to the location and characteristics of the implants used, since in many cases, these patients present limited ridges with deep concavities in the anterior mandible [19,20]. Although the number of implants has effect on the peri-implant stress distribution; it has been reported that a lower number of implants can slightly increase the stress in abutment and screws [17,18]; however, the use of frameworks and abutments may compensate these biomechanical weaknesses [17,18,19].

The present systematic review is justified by the scarcity of studies evaluating the clinical outcomes of three implants ISFCDPs. It set out to assess the clinical behavior of mandibular ISFCDPs by analyzing implant and prosthetic survival rates, marginal bone loss, biological/technical complications, and patient-reported outcomes.

## 2. Materials and Methods

This systematic review followed guidelines established in the PRISMA (Preferred Reporting Items for Systematic Review and Meta-Analyses) statement and is registered in the International Prospective Register of Systematic Reviews (PROSPERO) (Reg. No. CRD42020188102). It set out to answer the following PICO question (Population, Intervention, Comparison, and Outcome): “In edentulous mandibular patients rehabilitated with mandibular implant-supported fixed complete dental prostheses, is the use of three implants viable in terms of survival rates compared with more than three dental implants?”; whereby Population (P) was defined as mandibular edentulous patients; Intervention (I) as fixed prosthesis supported with three implants; Comparison (C) as mandibular implant-supported fixed complete dental prostheses with four or more dental implants; and the main Outcomes (O) measured were implant and prosthodontic survival rates (secondary outcomes were biological and technical complications and marginal bone loss).

### 2.1. Eligibility Criteria

This systematic review included randomized controlled trials (RCTs), clinical prospective and retrospective cohort studies, cross-sectional studies, case-control studies, and case reports.

#### 2.1.1. Inclusion Criteria

- Clinical human studies.
- Randomized controlled clinical trials, cohort studies, case-control studies, cross-sectional studies.
- Patient sample (related to the topic) of at least 10.
- Follow-up of at least 1 year.
- Articles published up to February 2021.

#### 2.1.2. Exclusion Criteria

- Case series and case reports.
- Animal studies.
- In vitro studies.
- Insufficient information on implant and/or prosthetic survival rates.

### 2.2. Type of Intervention and Comparisons

All the articles selected enrolled more than 10 patients rehabilitated with three mandibular implants supporting fixed complete dental prostheses, compared with 4, 5 or 6 implants supporting the same. Due to the lack of randomized clinical trials comparing three vs. more than three implants supporting mandibular fixed complete dental prostheses, the review included prospective or retrospective cohort studies of mandibular implant-supported fixed complete dental prostheses on three dental implants.

### 2.3. Sources and Search Strategy

An electronic search was conducted in the online databases PubMed, Web of Science, and Cochrane Library up to February 2021 for articles in English and Spanish language journals with no limit placed on publication date. In addition, manual searches were made in relevant prosthodontic and oral implantology journals, in the reference sections of articles identified in the electronic search, as well as a grey literature search in the Open Grey website.

The search strategy used for databases PubMed, Web of Science and Cochrane Library consisted of the following Medical Subject Headings (MeSH-Terms) and free terms: (edentulous) AND (dental prosthesis OR denture OR dental implant) AND (number OR (three OR four OR five OR six OR seven OR eight) AND implant) AND (survival rate OR complication OR outcome).

### 2.4. Study Selection and Screening Methods

Two reviewers (L.S.L. and P.M.M.) independently screened the titles and abstracts of the articles identified in searches. The same reviewers read the full manuscripts of those studies that fulfilled the inclusion criteria, or those with insufficient data in the title and abstract to reach a clear conclusion, before making a final selection. Any disagreement was resolved by discussion with a third reviewer (J.C.B-B.). Inter-reviewer reliability in the selection process and after full-text analysis was calculated to obtain the percentage of agreement and kappa correlation coefficient. If more than one study investigated the same patient cohort, the work with the longer follow-up period was selected for inclusion.

### 2.5. Data Collection and Items

The primary outcome was the clinical behavior of ISFCDPs supported by three dental implants in terms of implant and prosthetic survival rates. Secondary outcomes assessed were marginal bone loss, associated complications, and patient-reported outcomes. The same two reviewers performed duplicate data extraction. When data was incomplete or missing, the authors of the studies were contacted. If agreement could not be reached, data was excluded until further clarification was available. The data extracted were as follows: authors, year of publication, study design, number of patients, mean age, mean follow-up, number of implants, whether implants were angled or not, type of rehabilitation and loading, implant survival, prosthetic survival, patient satisfaction, complications, and marginal bone loss.

### 2.6. Quality Assessment in Individual Studies

Quality assessment was conducted using the Newcastle-Ottawa scale (NOS) for cohort studies. This scale includes three main categories: selection of study groups, comparability of participants, and outcome. Each individual study received a maximum of 9 points [21].

### 2.7. Statistical Analysis

Implant and prosthetic survival rates were defined as the implants and prostheses remaining in situ without modification for the entire follow-up period. Meta-analysis of observational studies was performed to determine implant survival, grouping the survival rates calculated by analyzing the cases in which implants were maintained during the follow-up period in each of the included studies with a 95% confidence interval using a fixed or random effects model depending on the heterogeneity of each study. The chi-square test and the Cochran’s Q test were applied to determine heterogeneity; if the chi-square test obtained between 0 and 50% and the *p*-value of the Q test was greater than 0.05, the level of heterogeneity was considered as within acceptable limits and so a fixed effects model was used. The same procedure was followed to calculate prosthetic survival. Analyses were performed using Stata version 15 software (Stata Corp., College Station, TX, USA).

### 2.8. Publication Bias

Publication bias was assessed visually by means of funnel plot analysis (symmetry or asymmetry) using Stata version 15.

## 3. Results

### 3.1. Study Selection

**Figure 1:**
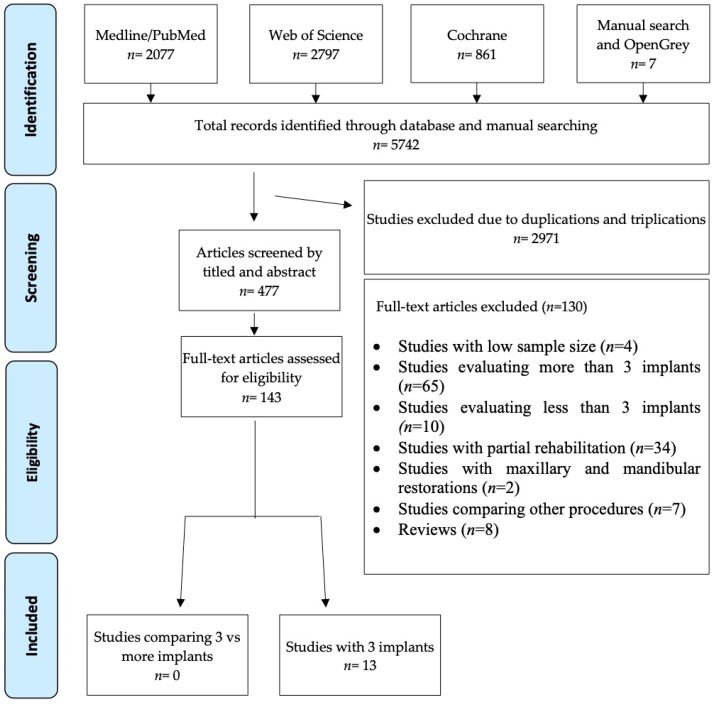
Flow chart of the electronic and manual screening and the selection process of studies.

The initial electronic database search yielded 5735 articles and the manual search identified seven more. Of these studies, 2971 were duplicates or triplicates and were removed. After an initial scanning to eliminate articles not relevant to the PICO question followed by title and abstract screening, a total of 143 articles were selected for full text analysis. Finally, a total of 13 studies were selected for data extraction, eight of them prospective studies and five retrospectives. The search and selection process are illustrated by the flow diagram shown in Figure 1.

### 3.2. Study Characteristics

Information about the studies included for review and results (study type, sample size, implant survival rate, follow up, rehabilitation type, loading, implant survival rate, prosthetic survival rate, patient-reported outcomes, complications, and marginal bone loss) are shown in Table 1 and Table 2. The 13 articles were all cohort studies, five retrospective and eight prospective. All patients attended follow-up periods of at least 1 year after dental implant placement and the maximum follow-up period was 6 years [22].

### 3.3. Synthesis of Results

#### 3.3.1. Inter-Investigator Agreement

Cohen’s Kappa statistic between the two reviewers (L.S.L. and P.M.M.) was 0.972 (CI 95% 0.972–0.972) for title and abstract selection and 0.986 (CI 95%: 0.988–0.983) for full text assessment, therefore, the agreement level was considered almost perfect. Third reviewer consensus evaluation was not necessary.

#### 3.3.2. Patient Characteristics

The 13 studies included 728 patients with a mean age of 63.89 years. Twelve studies recruited 351 men and 377 women, and one did not report patient sex. Out of a total of 728 patients, 2184 dental implants were placed to support 728 mandibular implant-supported screw-fixed prostheses (each supported by three implants). Nine studies placed the implants straight, three studies tilted posterior implants, and one study did not mention angulation [22].

Six studies mentioned the use of abutments [1,7,16,22,23,27]; nevertheless, only Hatano et al. [23] provided extra information about the abutment type (multiunit, minuscone or estheticone). Moreover, De Bruyn et al. [7] brought information about abutment height (varying between 3–5.5 mm).

#### 3.3.3. Implant and Prosthetic Survival Rates and Effects on Model Results

**Figure 2:**
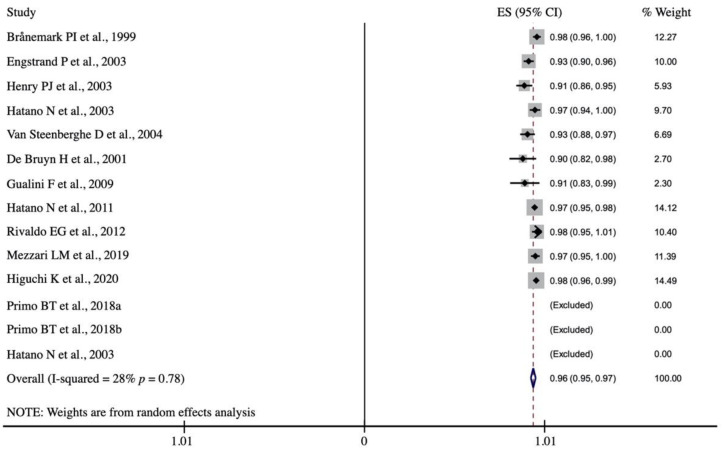
Forest plot. Implant survival rate after 1–6-year follow-up.

Quantitative analysis included observational studies comprising a total of 728 patients, who received 2184 implants; 89 implants failed, making an implant survival rate of 95.9% (95% CI: 94.6–97.3%; *p* = 0.001) after 1–6-year follow-up (Figure 2).

**Figure 3:**
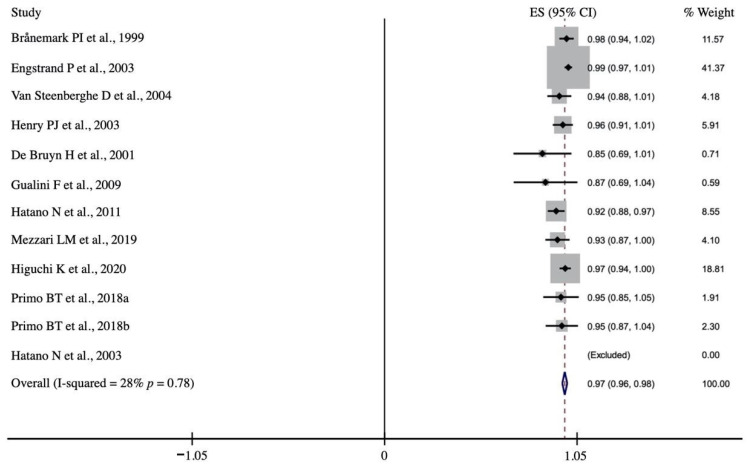
Forest plot. Prosthetic survival rate after 1–6-year follow-up.

Moderate statistical heterogeneity was detected between the groups, so a random effects model was applied [X^2^ = 22.94 (df = 10); *p* = 0.011; I^2^ = 56.4%]. The prosthetic survival rate was reported in 11 studies and was estimated to be 97.0% (95% CI: 95.7–98.3%; *p* = 0.001). Low heterogeneity was detected among the studies for this variable (X^2^ = 13.89 (df = 10); *p* = 0.178; I^2^ = 28.0%] (Figure 3).

#### 3.3.4. Antagonist Dentition and Prosthetic Loading Protocols

Antagonist dentition varied, with conventional complete dentures in three studies [1,26,28] and natural teeth, implants, or fixed tooth-supported restorations in seven studies [4,5,6,7,22,24,25]. Three studies did not report the type of antagonist dentition [23,27].

All the studies adopted immediate or early loading protocols; eight of them performed immediate loading exclusively (within 1–7 days), while the other five studies performed both immediate and early loading (within 1 week to 2 months) without distinguishing between the two [29].

#### 3.3.5. Denture Material and Torque of the Screwed-Retained Prostheses

All the manufactured ISFCDP were acrylic prosthesis with metal framework inside it, except the study of Ayna et al. [22], who delivered both: acrylic prosthesis with metal framework and metal-ceramic prosthesis.

Concerning the torque applied to fix the screw-retained prosthesis, only four studies provided this information [1,4,22,27] varying from 15–45 N.

#### 3.3.6. Marginal Bone Loss

Marginal bone loss was reported in six studies, varying between 0.13 and 1.62 mm after one year [4,7]. De Bruyn et al. reported 2.1 mm of marginal bone loss after a 3-year follow-up, while others reported 0.73–2.65 mm 5 years after loading [5,25,26]. The study with the longest follow-up [22] obtained 0.9–1 mm marginal bone loss after 6 years.

#### 3.3.7. Patient Reported Outcomes

Six studies did not register patient-reported outcomes [1,5,16,24,26,28]. One study simply asked patients if they were satisfied with treatment or not [23], two studies used the OHIP-14 and OHIP-Edent questionnaires respectively [22,27], and four studies reported varying satisfaction levels of 77–100% using other questionnaires [4,6,7,25].

#### 3.3.8. Biological and Technical Complications

Brånemark et al. did not report any complications; likewise, Van Steenbergue et al. and Rivaldo et al. did not mention complications [1,24]. The other 10 studies did not report major technical or biological complications, the most frequent minor complications being screw loosening, ranging from 1.6–16% [5,27] and tooth or veneer acrylic fractures.

#### 3.3.9. Quality Assessment of Individual Studies

The NOS scale is based on three categories: selection (S), comparability (C), and exposure (E). These sections are subdivided into nine different criteria: S1 = adequate case definition; S2 = representativeness of the cases; S3 = selection of controls; S4 = definition of controls; C1 = comparability of cases; C2 = controls based on the analysis; E1 = ascertainment of exposure; E2 = same method of ascertainment for cases and controls; and E3 = nonresponse rate. The nine criteria can receive a response of “yes” (1 point), “no,” or “cannot tell” (0 points for both). A classification of 7 to 9 points corresponds to a low risk of bias, 5 to 6 points to a medium risk of bias, and fewer than 5 points to a high risk of bias.

The assessed studies obtained scores of 6 points in six studies (medium bias) [1,5,6,7,16,23] and the remaining seven obtained 5 points (medium bias) [4,22,24,25,26,27,28]. These scores pointed to an adequate quality of evidence among the studies reviewed (Table 3).

#### 3.3.10. Publication Bias

**Figure 4:**
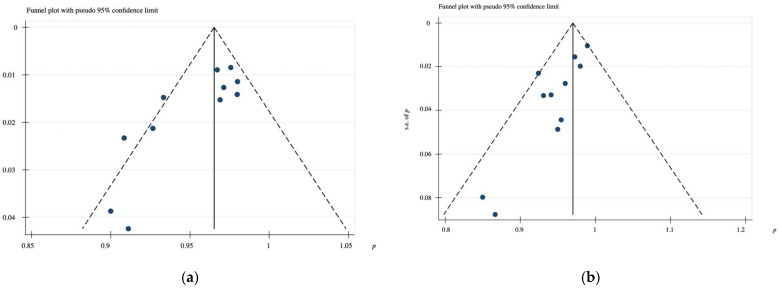
Funnel plot: (**a**) Implant survival rate after 1–6-year follow-up; (**b**) Prosthesis survival rate after 1–6-year follow-up.

The studies showed moderate symmetry in visual assessment of the funnel plot, indicating little publication bias (Figure 4).

## 4. Discussion

This systematic review and meta-analysis analyzed implant and prosthetic survival rates of mandibular ISFCDPs on three implants, as well as marginal bone loss, associated complications, and patient-reported outcomes.

Evidence for the optimal number of implants needed to support a fixed rehabilitation in edentulous patients is limited. Previous reviews that have investigated the ideal number of implants have reported a strong predilection for 4–6 implants, which is a well-documented treatment option obtaining high implant survival rates after 5-year (96.3–98.8%) and 10-year follow-up periods (92.8–97.7%) [30].

Eliasson et al. also concluded that four implants may be enough to support a fixed mandibular prosthesis, providing that these implants are at least 10 mm long [31]. Nevertheless, these reviews stressed the need for further clinical trials comparing this widely accepted option (4–6 implants) with 3 implants for fixed prostheses in the edentulous mandible [32,33], which the authors considered a potentially valid option.

In this context, the systematic review by De Luna Gomes et al. [34], which included studies with follow-up periods ranging from 5–15 years, set out to establish the optimal number of implants for implant-supported complete-arch prostheses, concluding that implant number did not influence implant or the prosthetic survival rates, technical complications, or marginal bone loss. Polido et al. obtained similar results for implant and prosthetic survival rates, comparing fixed complete-arch prostheses supported by five or more implants and with fewer than five implants [33].

Likewise, Moraschini et al. [35] obtained high survival rates (for both implants and prostheses), clinically acceptable marginal bone loss, and few technical or biological complications when mandibular fixed complete-arch prostheses were supported by 2–4 implants (90–100% implant survival and 93.7–100% prosthetic survival rate). Lima et al. evaluated mandibular implant-supported prostheses with different combinations of implants (3, 4 or 5), concluding that mandibular prostheses using three implants have shown a satisfactory implant survival rate and peri-implant bone loss during the first year of function. Meanwhile the prosthesis survival rate was inferior compared with mandibular profile prosthesis supported by a higher number of implants [36].

Multiple factors affect treatment planning and the number of implants used to support a mandibular ISFCDP. These include the patients’ wishes, their individual circumstances, the anatomical situation and the dental practitioner’s knowledge and experience; all will play a part in determining the type of treatment selected [19,20,37].

Rehabilitation of the edentulous mandible with three implants can reduce the economic costs of surgical and prosthetic phases, and so make treatment more acceptable to the patient [19]. Notwithstanding, and according to scientific literature, the number of implants have effect on the peri-implant stress distribution, reporting that a lower number of implants can slightly increase the stress in abutment and screws [17,18]. However, and taking into account the obtained results, the use of three dental implants for mandibular full-arch fixed dental prostheses showed similar outcomes and complication rates in comparison with mandibular ISFCDP on more than three implants [30,31,32,33].

The studies included in this systematic review included a total of 728 patients, who received 2184 dental implants. A mean survival implant rate of 95.9% (95% CI: 94.6–97.3%) and a prosthetic survival rate of 97.0% (95% CI: 95.7–98.3%) were obtained after at least 1 year, up to 6 years. As far as the authors are aware, only one previous systematic review has focused solely on fixed mandibular prostheses supported by three implants [38], and it should be noted that the review did not include antagonist dentition or patient-reported outcomes. The results were similar to the present review in terms of implant survival rates (95.43%), although differences were found in prosthetic survival rates (89.68% versus 97%). This may be partly due to the different eligibility criteria applied and the statistical data processing methods used (meta-analysis).

Regarding antagonist dentition, due to the heterogeneity of the studies reviewed, rigorous comparison was not possible but given the generally favorable data obtained, it may be deduced (with caution) that mandibular ISFCDP supported by three implants can be considered a viable option regardless of antagonist dentition.

Marginal bone loss is considered an important parameter when evaluating the success of implant-based dental rehabilitations. It is normal for vertical marginal bone loss around implants to reach a maximum of 1.5 mm to 2 mm during the first year of functional loading [39,40,41]. Although the marginal bone loss results reported in this review fell within acceptable limits, these data should be interpreted with caution, as the studies analyzed presented considerable heterogeneity in terms of implant angulation and the duration of follow-up periods.

Another significant aspect for consideration is patients’ own perception of their oral health status and how it impacts on daily life and on quality of life. In this sense, in the studies reviewed, patient reported outcomes obtained acceptable scores, although the studies did not include any comparison groups.

Patient-reported outcomes are negatively influenced by any biological and technical complications that occur, which may prolong treatment time and involve unexpected additional expense [42]. The studies reviewed did not report any complications such as peri-implant diseases or implant fractures. Regarding other technical complications, the prosthetic survival rates concur with other studies of implant-supported fixed prostheses in edentulous mandibles using larger numbers of dental implants [35,43,44], with the most frequent complication being screw loosening and tooth veneer acrylic fractures.

The present systematic review had some limitations, particularly the heterogeneity of the studies analyzed and the lack of randomized controlled clinical trials comparing two groups of mandibular ISFCDPs (three implants compared with larger numbers of implants). In addition, the studies reviewed had different medium- and long-term follow-up periods, while some did not provide sufficiently clear information about the duration of follow-up periods. Moreover, some of the studies included both tilted and straight implants with immediate and delayed loading protocols, which could have affected implant and prosthetic survival rates, results, and complication rates.

## 5. Conclusions

Within the limitations of this systematic review, it may be concluded that mandibular implant-supported fixed complete dental prostheses on three dental implants would appear to be a reliable option for restoring edentulous patients, obtaining an implant survival rate of 95.9% and a prosthetic survival rate of 97.0% after 1–6-year follow-up. The results presented acceptable rates of marginal bone loss and biological/technical complications and satisfactory patient-reported outcomes that were similar to the results obtained with mandibular implant-supported fixed complete dental prostheses on more than three implants.

These findings should be interpreted with caution as multiple factors contribute to successful treatment planning and determine the number of implants used to support a mandibular ISFCDP. Further comparative studies are needed to confirm the viability of mandibular ISFCDPs supported by three implants, with adequate protocols, along with sufficient sample sizes and follow-up periods.

## Figures and Tables

**Table 1:** Information about studies reviewed, evaluating ISFCDPs on 3 dental implants: study type, sample size, implant survival rate, follow-up, rehabilitation type, loading.

Author and Year	Study	Patient Number	Implant Number Straight/Tilted	Follow-Up	Rehabilitation Type	Loading	Antagonist Dentition
Brånemark et al., 1999 [4]	Prospective Mandible	50 24 females/26 males MA: 64 years (45–86)	150 Straight	75 Mo PIP-50 patients at 3 Mo, 49 at 6 months, 42 at 12 Mo, 13 patients at 24 Mo and 3 at 36 Mo	Screw 12 pieces	Immediate loading (7 h)	34 patients’ maxillary dentures, 11 tooth-supported bridges, 5 implant-supported reconstructions)
Engstrand et al., 2003 [5]	Prospective Mandible	95 42 females 53 males MA: 68.5 years (45–89)	285 Straight	12–70 Mo (mean 30 Mo) 98.9% of patients 12 Mo, 68.4% 12 Mo, 49.5% 36 Mo, 22.1% 48 Mo, 9.5% 70 Mo.	Screw 12 pieces	67.4% immediate loading/ 6.3% the next 1 or 2 days/ 2.3% in the next 3–10 days/ 6.3% in the 11–20 days/ 11.6% in 21–30 days3.2% in 31–40 days	56 patients’ removable complete dentures, 30 natural teeth or crowns/bridges, 9 patients implant-supported prostheses
Henry et al., 2003 [6]	Prospective Mandible	51 23 females 28 males MA: 62.3 years(43–79)	153 Straight	12 Mo (49 patients)	Screw NM	76% of patients immediate loading (same day)/ 24% in 2 or more days after surgery	>5 patients’ natural teeth + tissue-integrated prostheses, complete denture, overdenture, conventional removable prostheses, 5 patients’ natural teeth + removable prostheses, tissue-integrated prostheses, conventional bridge
Hatano et al., 2003 [23]	Retrospective Mandible	43 17 females 26 males MA: 61.7 years(48–82)	129 Straight	3–49 Mo	Screw NM	Immediate loading (same day)	NM
Van Steenberghe et al., 2004 [24]	Prospective Mandible	50 25 females 25 males MA: 56.5 years(45–80)	150 Straight	12 Mo (45 patients)	Screw NM	Immediate loading in 34 patients/ One patient rehabilitated after 10 days/ 15 patients after 1–3 days	38 maxillaries edentulous, 3 implants and bridge, 3 bridge on natural teeth, 6 dentate
De Bruyn et al., 2001 [7]	Prospective Mandible	20 12 female 8 males MA: 64 years (41–80)	60 Straight	36 Mo (15 patients 12 Mo follow-up, 5 patients 18 Mo follow-up)	Screw 10–12 pieces	Immediate loading	10 remaining natural or denture teeth
Gualini et al., 2009 [25]	Retrospective Mandible	15 4 females 11 males MA: 63.5 years (55–78)	45 Straight	60 Mo (15 patients at 12 Mo, almost 15 42–72 Mo)	Screw 12 pieces	Immediate loading (same day or the following day)	6 patients maxillary complete prostheses, 3 implant-supported constructions, 4 patients dentate, 2 patients partial dentures)
Hatano et al., 2011 [16]	Retrospective Mandible	132 67 females 65 males MA: 62.6 years (35–85)	396 Distal tilted	60 Mo (132 patients 12 Mo, 77 patients 60 Mo 3 patients at 120 Mo)	Screw 12–14 pieces	Immediate loading (same day)	NM
Rivaldo et al., 2012 [1]	Retrospective Mandible	33 64.2% females 35.8% males 38–83 years majority (43%) 51–60 years	99 Straight	18 Mo (33 patients)	Screw NM	Until 72 h after implant placement	Maxillary complete denture
Mezzari et al., 2019 [26]	Retrospective Mandible	58 35 females 23 males MA: 63.3 ± 7.9 years (46–81 years)	174 NM	60 Mo	Screw NM	Immediate loading	Maxillary complete dentures
Ayna et al., 2020 [22]	Prospective Mandible	29 13 females 16 males MA: 65 ± 6 years	87 Tilted distal implants	72 Mo	Screw 12 pieces	Immediate loading (first 24 h)	NM
Higuchi et al., 2020 [27]	Prospective Mandible	110 60 females 50 males MA: 61.7 years	330 Straight	12 Mo (104 patients went to follow up)	Screw NM	76.4% immediate loading (1–2 days) 23.6% early loading (3–10 days)	NM: stable opposing dentition
Primo et al., 2018 [28]	Prospective double-arm Mandible	20 patients 16 females 4 males MA: 64 years 22 patients 18 females 4 males MA: 73.5 years	60 Distal tilted 66 Distal tilted	18 Mo	Screw NM	Immediate loading Early loading	Complete maxillary dentures

MA: medium age. PIP: post-implant placement. Mo: Months. NM: Not Mentioned.

**Table 2:** Information about selected studies evaluating ISFCDPs on 3 dental implants, including implant and prosthetic survival rate, patient-reported outcomes, complications and marginal bone loss.

Author and Year	Implant Survival	Prosthetic Survival	Patient Satisfaction	Complications	Marginal Bone Loss
Brånemark et al., 1999 [4]	98% (3 failures)	98%	94% 20 questions questionnaire 10 days post-operative	No complications	0.13 mm first year (42 patients) 0.26 mm second year (13 patients) 0.53 mm third year (3 patients)
Engstrand P et al., 2003 [5]	95% first year 93.3% third year 93.3% fifth year (18 failures)	99%	NM	Dehiscence in 3 patients Transient paresthesia in 5 patients Mucosal inflammation in three patients 2 screw fractures, loosening of 16% of patients	0.73 mm first year 0.73 + 0.16 mm second year 0.73 + 0.16 + 0.13 mm third, fourth and fifth year
Henry et al., 2003 [6]	96.1% 3 months 95.4% 6 months 90.7% first year (14 failures)	96.1% 3 months 96.1% 6 months 94% first year	97% of patients did not have phonetic problems 87% did not have masticatory problems 87% did not have aesthetic problems 76% felt rehabilitation as own 100% of patients would repeat the surgery	1 patient with paresthesia 11 patients with prosthesis bar loosening 2 patients with prosthesis loosening 2 patients with prosthesis fracture 1 patient with mucositis	0.4 mm
Hatano et al., 2003 [23]	97.6% SUCCESS (3 failures)	100%	Patients were satisfied, No questionnaire	1 patient with screw loosening 1 patient structure did not fit	NM
Van Steenberghe et al., 2004 [24]	92.7% (11 failures)	95%	NM	NM	1.1 mm with periapical 0.8 mm and 0.7 mm in vestibular and lingual in CBCT 1-year follow-up
De Bruyn et al., 2001 [7]	90.5% (6 implants, 1 before loading, was replaced)	85%	87% were satisfied at 3 months 77% of satisfaction at year	1 patient pain after implant surgery fracture of temporary cylinder in a patient with a provisional prosthesis 1 abutment fracture	1.6 mm at one year 2.1 mm at three years
Gualini et al., 2009 [25]	91.1%(4 failures)	86.7%	100% of patients satisfied with prosthesis function 2/13 not completely satisfied with aesthetics	6 screw loosening, 12 resin or tooth fractures (90% occurred in 3/13 patients), 6 need for upper bar modification	0 mm in 11 patients 0.1 mm 1 patient 0.5 mm 1 patient 5 years follow-up
Hatano et al., 2011 [16]	96.7% (13 failures)	92.4%	NM	Acrylic tooth fracture and occasional loss of Access-hole fillings. No major prosthetic complications	NM 5 years follow-up
Rivaldo et al., 2012 [1]	97.97% (2 failures)	NM	NM	NM	0.66 ± 0.51 mm for left implant 0.92 ± 0.61 mm for middle implant 0.82 ± 0.52 mm for right implant 18 months follow-up
Mezzari et al., 2019 [26]	97.13% (5 failures)	93.1% SUCCESS	NM	27 prosthetic complications: 10 patients cover screw issues, 7 loosening of the prosthetic screw, 13 torque loss or insufficient torque of the abutment, 5 issues with the acrylic portion of the prosthesis, 8 unsatisfactory occlusion 50% of patients experienced at least one prosthetic complication	2.65 ± 1.06 mm middle implant 2.11 ± 0.84 mm distal implants 5 years
Ayna et al., 2020 [22]	100%	NM 14 acrylic resin prosthesis with titanium framework and 15 patients received metal-supported ceramic restoration	OHIP-14 and masticatory forceSatisfaction when immediate loading	6 patients with supra-structure fracture (four in canines repaired in clinic, 2 reached metal framework and were repaired in laboratory). 2 patients with superficial veneer fractures repaired in situ.	0.9 ± 1 mm for left implant 1 ± 1 for right implant 0.9 ± 1 mm for middle implant 6 years
Higuchi et al., 2020 [27]	97.5% (8 failures)	97.3%	OHIP EDENT-21 Function and esthetics great results from prosthesis loading until a year of follow-up	42 adverse events Transient sensitive alteration in 8 patients, 5 patients with persistent pain after surgery, submandibular swelling on the right side in 2 patients, 1.9% of screw loosening, 6 tooth chipping3 tooth fracture	0.62 ± 1.39 mm 1 year
Primo et al., 2018 [28]	98.33% (1 failure) 98.49% (1 failure)	95.23% 95.65%	NM	9 cover screw issues, 6 patients with acrylic portion problems, 5 patients with occlusion unsatisfactory, plaque in 29 implants, prosthesis hygiene unsatisfactory in 9 patients 31 implants with plaque presence, unsatisfactory prosthesis hygiene in 8 patients	1.38 mm for right implant, 1.73 mm for middle implant and 1.56 mm for left implant 1.58 mm for right implant, 1.72 mm for middle implant and 1.53 mm for left implant 18 months

**Table 3:** Quality assessment of studies reviewed according to the Newcastle-Ottawa scale.

Study	Selection				Comparability		Outcome			Number of Stars (Out of 9)
	S1	S2	S3	S4	C1	C2	E1	E2	E3	
Brånemark et al., 1999	★	0	★	★	★	0	0	★	0	5
Engstrand et al., 2003	★	0	★	★	★	0	★	★	★	6
Henry et al., 2003	★	0	★	★	★	0	★	0	★	6
Hatano et al., 2003	★	0	★	0	★	0	★	★	★	6
Van Steenberghe et al., 2004	★	0	★	★	★	0	0	0	★	5
De Bruyn et al., 2001	★	0	★	★	★	0	★	★	0	6
Gualini et al., 2009	★	0	★	0	★	0	0	★	★	5
Hatano et al., 2011	★	0	★	0	★	0	★	★	★	6
Rivaldo et al., 2012	★	0	★	★	★	0	0	★	★	6
Primo et al., 2018	★	★	0	★	★	0	0	★	0	5
Mezzari et al., 2019	★	0	★	0	★	0	★	★	0	5
Ayna et al., 2019	★	0	★	★	★	0	0	★	0	5
Higuchi et al., 2020	★	0	★	★	★	0	0	0	★	5

Each star (★) counted as one point for each category

## Data Availability

The data that support the findings of this study are available from the corresponding author, J.C.-C.B., upon reasonable request.
